# Racial and ethnic difference in the risk of fractures in the United States: a systematic review and meta-analysis

**DOI:** 10.1038/s41598-023-32776-1

**Published:** 2023-06-10

**Authors:** Yueyang Bao, Yingke Xu, Zhuowei Li, Qing Wu

**Affiliations:** 1 Nevada Institute of Personalized Medicine, College of Sciences, University of Nevada, Las Vegas, NV USA; 2 Department of Biology, McMaster University, Hamilton, ON L8S 4L8 Canada; 3 Department of Epidemiology and Biostatistics, School of Public Health, University of Nevada, Las Vegas, NV USA; 4 Department of Biomedical Informatics, Center for Biostatistics, The Ohio State University, Columbus, OH USA

**Keywords:** Epidemiology, Bone

## Abstract

This systematic review and meta-analysis examined the association between race and ethnicity and fracture risk in the United States. We identified relevant studies by searching PubMed and EMBASE for studies published from the databases’ inception date to December 23, 2022. Only observational studies conducted in the US population that reported the effect size of racial-ethnic minority groups versus white people were included. Two investigators independently conducted literature searches, study selection, risk of bias assessment, and data abstraction; discrepancies were resolved by consensus or consultation of a third investigator. Twenty-five studies met the inclusion criteria, and the random-effects model was used to calculate the pooled effect size due to heterogeneity between the studies. Using white people as the reference group, we found that people of other races and ethnic groups had a significantly lower fracture risk. In Black people, the pooled relative risk (RR) was 0.46 (95% confidence interval (CI), 0.43–0.48, *p* < 0.0001). In Hispanics, the pooled RR was 0.66 (95% CI, 0.55–0.79, *p* < 0.0001). In Asian Americans, the pooled RR was 0.55 (95% CI, 0.45–0.66, *p* < 0.0001). In American Indians, the pooled RR was 0.80 (95% CI, 0.41–1.58, *p* = 0.3436). Subgroup analysis by sex in Black people revealed the strength of association was greater in men (RR = 0.57, 95% CI = 0.51–0.63, *p* < 0.0001) than in women (RR = 0.43, 95% CI = 0.39–0.47, *p* < 0.0001). Our findings suggest that people of other races and ethnic groups have a lower fracture risk than white people.

## Introduction

As the population in the United States ages, osteoporotic fractures are becoming an increasing public health concern^1^. By 2025, the annual economic burden of osteoporotic fractures is expected to exceed $25 billion, and its annual aggregate incidence is expected to surpass 3 million^2^. In recent years, the prevalence of osteoporosis among US adults aged 50 and over was 12.6%, and the prevalence of osteopenia, which can often progress to osteoporosis, was 43.1%^3^. These affected populations, which constitute a major proportion of the US population, are especially vulnerable to osteoporotic fractures. Patients discharged after suffering such fractures are still at an increased risk of subsequent fractures, morbidity, and mortality^4–6^.

These fracture rates and related mortality vary by race and ethnicity^7,8^. In white men and women, the incidence of hip fractures has decreased since 2000^9,10^. However, a similar decrease was not observed in Black people, Asian Americans, Hispanics, and American Indians^9,10^. Such difference could be due to health disparities existing across these groups with regard to osteoporosis awareness, in addition to disparities in osteoporosis screening, diagnosis, and treatment^11–13^. Given the current trends in demographic changes, populations from racial-ethnic minority groups are expected to compose a majority of the population growth in the following decades^14^. Understanding the association between race and ethnicity and fracture risk is vital in preventing fracture and decreasing the burden on racial and ethnic minorities and the healthcare system.

Recent literature has questioned the use of race and ethnicity in medicine, especially in the context of clinical algorithms, whose outputs are adjusted based on a patient’s race or ethnicity^15–17^. Often, these adjustments tend to underestimate the needs of minority patients, which may delay necessary interventions and further exacerbate racial disparities in medical care^16,17^. In the setting of osteoporosis, extensive research into the relationship between race and ethnicity and fracture risk has been scarce. Previous literature reviews addressing the issue have demonstrated that race and ethnicity do play a role in the risk of fracture in minority groups^18,19^. However, such reviews did not provide quantitative evidence about the association between race and ethnicity and the risk of fractures. These reviews were published over a decade ago and were not able to include recent reports^20–24^. To our knowledge, there have not been any systematic reviews or meta-analyses conducted on this subject. In light of these considerations, this study aimed to quantitatively assess all available studies to investigate the association between race and ethnicity and the risk of fractures, which is vital to promoting race-based methodologies for effective osteoporosis prevention and treatment.

## Results

### Literature search

**Figure 1:**
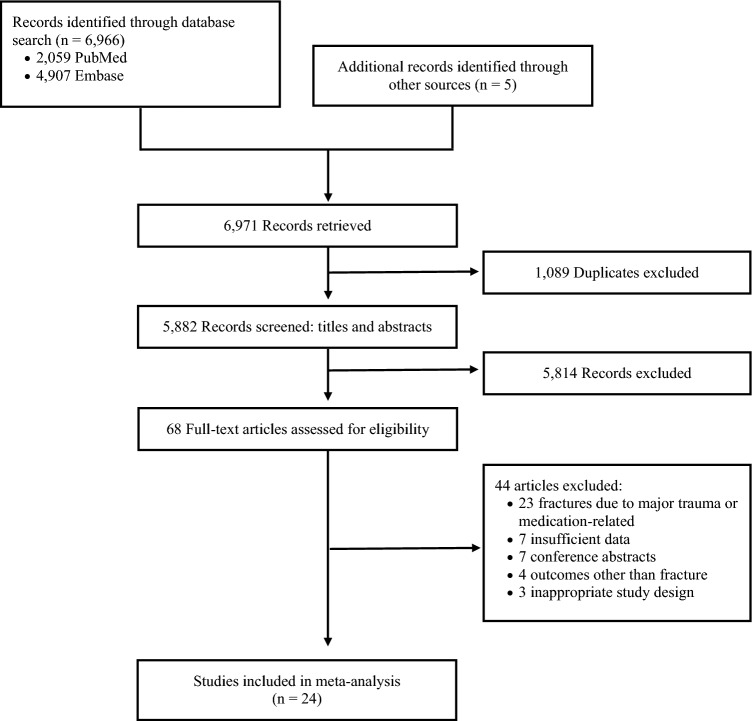
Study selection flow chart for meta-analysis.

From the initial literature search, we identified 6971 articles, as well as an additional 5 through searching references of potentially relevant articles (Fig. 1). After removing duplicates, 5882 articles were screened through their titles and abstracts, and 68 full-text articles were retrieved and assessed for eligibility. At this initial screening stage, the inter-rater agreement was fair (κ = 0.32). From these, 25 articles met the inclusion criteria^10,20–43^. However, two studies were conducted by the same research team and used the same data source but reported different outcomes^33,34^. These two studies were combined, and a final twenty-four studies were included in our meta-analysis. At this second stage, the inter-rater agreement was substantial (κ = 0.70).

### Study characteristics

The study characteristics of the twenty-four included studies are summarized in Table 1, which included a total of 7,234,903 participants. Of these twenty-four studies, one was case-control in design, four were cross-sectional, and nineteen were cohort. The majority of the studies (13/24) included participants ≥ 65 years, with the range of inclusion age being ≥ 17 to ≥ 70 years. Twenty-two studies reported fracture risk for Black people, eleven for Hispanics, nine for Asian Americans, and six for American Indians. Eleven studies reported strictly hip fractures as the outcome, 3 reported strictly vertebral fractures as the outcome, and ten reported various fractures as the outcome. With regards to the variables adjusted, three did not adjust for any variables, eight adjusted solely for age, and thirteen adjusted for age and other variables.

**Table 1:** Characteristics of twenty-four studies on the association of race and risk of fractures.

Study	Study population	Study design	Races studies	Outcomes	Outcome assessment	Variables controlled
Bauer et al., 1986^51^	80 cases and 160 controls aged ≥ 50 years from a hospital in San Antonio, Texas	Case–control	Black people, Hispanics	Hip fracture	Medical records	N/A
Bauer et al., 1987^52^	822 women aged ≥ 15 years from a hospital’s walk-in clinic	Cross-sectional	Black people, Hispanics	Vertebral fracture	Roentgenographic reports	Age, history of trauma in the past three months, use of steroids, abuse of alcohol or drugs
Jacobsen et al., 1990^53^	745,435 patients aged ≥ 65 years from the HCFA and the Department of Veterans Affairs	Retrospective cohort	Black people	Hip fracture	ICD-9-CM codes from discharge records	Age
Fisher et al., 1991^54^	22,039 men and women aged ≥ 65 years from New England Medicare enrollees	Retrospective cohort	Black people	Hip fracture	ICD-9-CM or CPT Version 4 codes	Age, sex, nursing home residence, and comorbidity index, and all their two–three, and four-way interaction
Griffin et al., 1992^37^	6802 men and women aged ≥ 65 years among Tennessee Medicaid enrollees	Retrospective cohort	Black people	Nonvertebral fracture	Computer algorithm	Age, race, sex, nursing home residence
Baron et al., 1994^38^	50,998 patients aged ≥ 65 years from standard 5% sample of Medicare population maintained by HCFA	Retrospective cohort	Black people	Hip fracture, distal forearm fracture, proximal humerus fracture, and ankle fracture	ICD-9-CM or CPT Version 4	Age, race, gender, and interaction between gender & race
Ross et al., 1995^39^	839 women aged ≥ 50 years from the Hawaii Osteoporosis Study and 762 women aged ≥ 50 from the Rochester Epidemiology Project	Prospective cohort	Asian Americans	Vertebral fracture	Spinal radiographs	Age
Karagas et al., 1996^40^	34,243 patients aged ≥ 65 years from a 5% random sample of Medicare recipients	Retrospective cohort	Black people	Hip fracture	ICD-9-CM or CPT Version 4 codes	Age
Lauderdale et al., 1997^41^	58,598 men and women aged ≥ 65 years among a 50% Medicare sample	Retrospective cohort	Asian Americans	Hip fracture	ICD-9-CM codes	Age
Turner et al., 1998^42^	958 women aged ≥ 50 years from NHANES III, Phase 1	Cross-sectional	Black people, Hispanics	Hip fracture	Household interviews	Age, BMI, mothers' osteoporosis diagnosis, mothers' hip fracture status, physical activity, smoking status, alcohol use, dairy produce use
Bohannon et al., 1999^43^	2590 women aged ≥ 65 years from the Duke Established Populations for Epidemiologic Studies of the Elderly	Prospective cohort	Black people	Nonvertebral fracture	Household interviews by trained interviewers	Age, education, family income, residence, tobacco use, alcohol consumption, overweight at age 50 years, underweight at age 50 years, history of stroke, ambulation assistance required, activities limited because of health, cognitive impairment, depression, Rosow-Breslau limitations, chronic health problems, vision problems, subjective health, number of outpatient visits in the past year, diuretics, phenytoin, prednisone, thyroid supplements, calcium supplements, estrogen
Young et al., 2001^44^	7527 participants aged ≥ 70 from the Longitudinal Study on Aging	Prospective Cohort	Black people	Hip fracture	ICD-9-CM codes from discharge records	Age, gender, race, history of fall in the past year, exercise, attending church services past 2 weeks, hospitalization in the past year, BMI quartile, living arrangement, ADL and IADL limitations
Barrett-Connor et al., 2005^47^	197,848 women aged ≥ 50 years from the National Osteoporosis Risk Assessment	Prospective Cohort	Black people, Hispanics, Asian Americans, American Indian	Osteoporotic fractures (hip, rib, wrist, forearm, spine)	Mailed set of questionnaires; validated by telephone	Age, education, current health status, years since menopause, weight, estrogen use, cortisone use, BMD site/device
Tracy et al., 2006^48^	542 men aged ≥ 65 years from the Baltimore Men’s Osteoporosis Study	Prospective Cohort	Black people	Vertebral fracture	Radiographs	Age
Cauley et al., 2007^49^	159,579 women aged ≥ 50 years from the Women’s Health Initiative	Prospective Cohort	Black people, Hispanics, Asian Americans, American Indians	Any fractures (except fingers, toes, face, skull, or sternum)	Radiology reports for hip fractures and self-reports confirmed by physician review of medical records for non-hip fractures	Age, years since menopause, education, living with a partner, height, weight, caffeine intake, smoking, fracture history, parental fracture history, falls, current HT use, corticosteroid use, sedative/anxiolytics use, arthritis, depression, health status, parity
Mackey et al., 2007^50^	1446 patients aged ≥ 70 years from the Health, Aging, and Body Composition Study	Prospective Cohort	Black people	Nonvertebral	Self-reported at clinic visits and interviews; confirmed by medical documentation, including reviewing radiology report	Age
Cauley et al., 2005^45^ & 2008^46^	7970^45^ and 8332^46^ women aged ≥ 65 years from the Study of Osteoporotic Fractures	Prospective cohort	Black people	Non-vertebral fracture^45^,Vertebral fracture^46^	Letter or telephone every 4 months; confirmed by radiographic report	Age, femoral neck BMD, body weight, height, fracture since age 50 years, walking as a form of exercise, current calcium supplement use, current hormone therapy use, alcohol consumption in the past 30 days, diagnosis of osteoarthritis, diagnosis of COPD, fallen 2 or more times in the past year, use arms to stand up from a chair, current smoking^45^; Age, femoral neck BMD, body weight, height, grip strength, uses arms to stand, walks for exercise, current calcium supplements, past and current hormone use, health status, difficulty with ≥ 1 IADL, fracture history, diabetes, COPD^46^
Wright et al., 2012^55^	821,475 women and 632,162 men aged ≥ 65 years from random 5% sample of Medicare beneficiaries	Retrospective cohort	Black people, Asian Americans, Hispanics	Hip fracture	ICD-9	Age
Looker et al., 2013^20^	2743 men and women aged ≥ 65 years from NHANES III	Cross-sectional	Black people, Hispanics	Osteoporotic fractures (hip, radius, spine, humerus)	ICD-9, HCPCS, or CPT codes	Age, sex, height, weight, education, current smoking, use of bone-enhancing drugs, self-reported physician's diagnosis of arthritis, femur neck BMD
Sullivan et al., 2016^10^	317,677 patients aged ≥ 55 years from all California Office of Statewide Health and Planning and Development non-federal hospital admissions	Retrospective cohort	Black people, Hispanics, Asian Americans, American Indians	Hip fracture	ICD-9-CM procedure codes	Age
Chang et al., 2016^21^	344,488 women aged ≥ 18 years from the Women’s Health Evaluation Initiative Master Database	Cross-sectional	Black people, Hispanics, Asian Americans, American Indians	Any fractures	ICD-9-CM codes	Age, residence, primary care visits, mental health clinic visits, service-connected disability rating
Berry et al., 2016^22^	892,837 men and women aged ≥ 65 years from a 100% sample of Medicare Part A claims nursing home residents	Retrospective cohort	Black people, Hispanics, Asian Americans, American Indians	Hip fracture	ICD-9 codes	N/A
Amir et al., 2019^23^	1,136,262 men and women aged ≥ 65 years from Medicare fee-for-service nursing home residents	Retrospective cohort	Black people, American Indians	Hip fracture	ICD-9 codes	Age, sex, medication, and clinical covariates
Yusuf et al., 2020^24^	1,780,451 men and women aged ≥ 67 years from a 20% Medicare database	Retrospective cohort	Black people, Hispanics, Asian Americans	Osteoporotic fractures	ICD-9-CM diagnosis code and/or CPT fracture repair procedure code	N/A

N/A = not available; HCFA = Health Care Financing Administration; ICD-9-CM = International Classification of Diseases—Ninth Revision—Clinical Modification; CPT = Current Procedural Terminology; NHANES III = Third National Health and Nutritional Examination Survey; BMI = body mass index; ADL = activities of daily living; IADL = instrumental activities of daily living; BMD = bone mineral density; COPD = chronic obstructive pulmonary disease; HT = hormone therapy; ICD-10 = International Classification of Diseases—Tenth Revision; HCPCS = Healthcare Common Procedure Coding System.

### Meta-analysis

**Figure 2:**
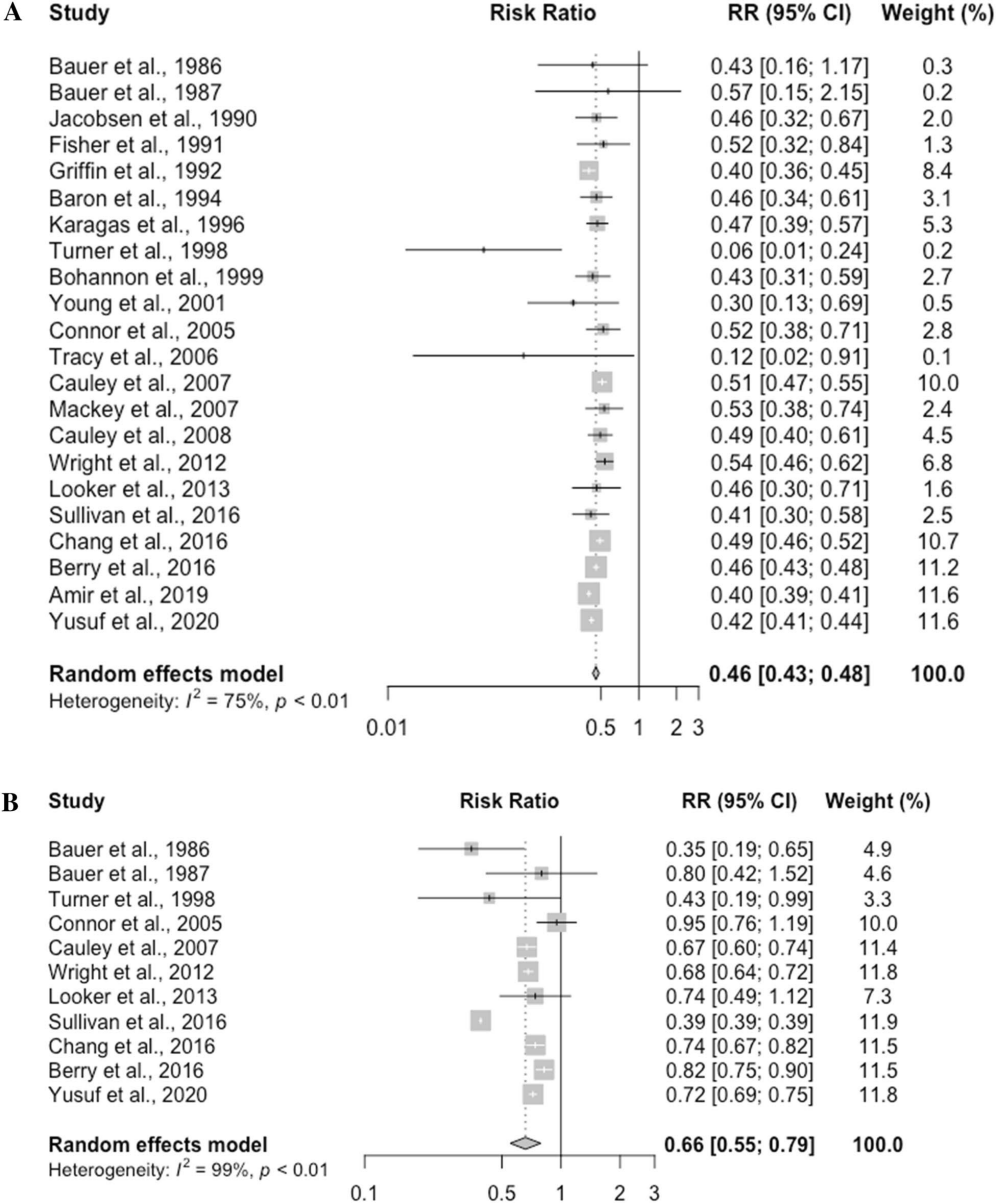

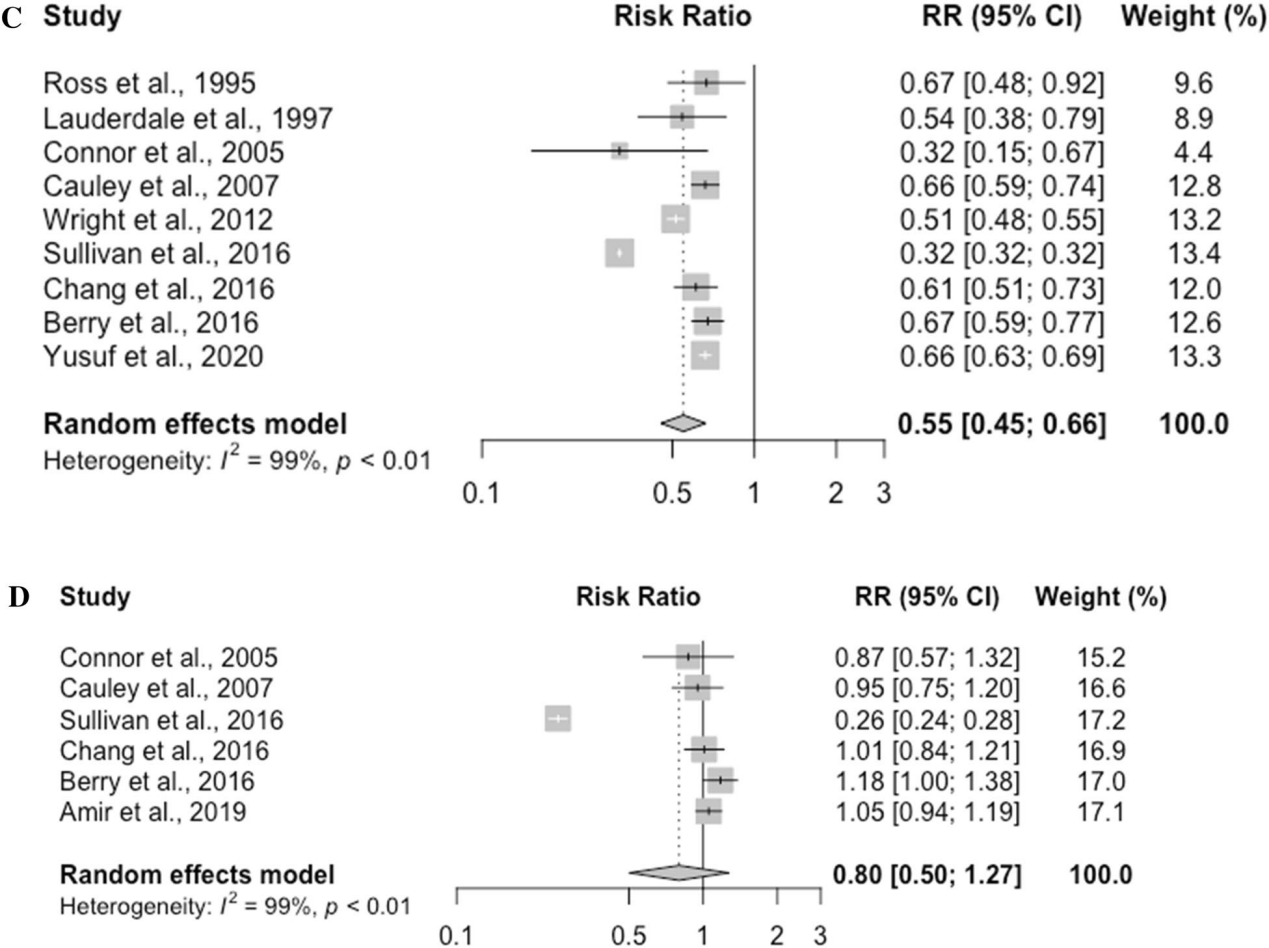
(**A**) Risk of fracture associated with Black race. (**B**) Risk of fracture associated with Hispanic ethnicity. (**C**) Risk of fracture associated with Asian race. (**D**) Risk of fracture associated with American Indian race.

The RRs of fracture risk in different races and ethnic groups are presented in Fig 2. Black people, Hispanics, Asian Americans, and American Indians have significantly lower fracture risk when compared to white people. The relative risk of fracture in Black people was 0.46 (95% CI, 0.43–0.48, *p* < 0.0001). The relative risk of fracture was 0.66 (95% CI, 0.55–0.79, *p* <0.0001) in Hispanics, 0.55 (95% CI, 0.45–0.66, *p* <0.0001) in Asian Americans, and 0.80 (95% CI, 0.41–1.58, *p* = 0.3436) in American Indians.

When different exclusion criteria were applied to the studies, with the exception of American Indians, the association between race and fracture risk changed slightly but remained significant. After applying various criteria, the RR ranged from 0.44 to 0.46 in Black people (Table 2). In studies investigating fracture risk in Hispanics, applying different exclusion criteria led to RR varying from 0.63 to 0.74 (Table 3). In studies that reported fracture risk in Asian Americans, the exclusion of studies according to different criteria resulted in RR ranging from 0.50 to 0.66 (Table 4). In American Indian studies, including studies that reported osteoporotic fractures only decreased the RR to 0.72. When other exclusion criteria were applied, the RR increased to the range of 1.02 to 1.10 (Table 5). However, the results in American Indians remain statistically insignificant after sensitivity analyses due to their wide 95% CIs including 1, and their *p*-values > 0.05.

**Table 2:** Relative risk of fracture associated with Black race according to different exclusion criteria.

Studies included	Studies (*n*)	Relative risk (95% CI)	*p*
All studies	22	0.46 (0.43–0.48)	< 0.0001
Studies that used osteoporotic fracture as the outcome^a^	15	0.45 (0.42–0.50)	< 0.0001
Studies that controlled for multiple fracture risk factors^b^	13	0.45 (0.41–0.50)	< 0.0001
Studies with participants aged ≥ 65 years only^c^	15	0.44 (0.42–0.47)	< 0.0001
Studies with methodological quality score ≥ 7^d^	13	0.46 (0.42–0.49)	< 0.0001
Studies that used HR for effect size^e^	7	0.45 (0.41–0.49)	< 0.0001

CI = confidence interval.

^a^Excludes Griffin et al. (1992)^37^, Baron et al. (1994)^38^, Bohannon et al. (1999)^43^, Cauley et al. (2007)^49^, Mackey et al. (2007)^50^, Chang et al. (2016)^21^, and Yusuf et al. (2020)^24^.

^b^Excludes Bauer et al. (1986)^51^, Jacobsen et al. (1990)^53^, Karagas et al. (1996)^40^, Tracy et al. (2006)^48^, Mackey et al. (2007)^50^, Sullivan et al. (2016)^10^, Wright et al. (2012)^55^, Berry et al. (2016)^22^, and Yusuf et al. (2020)^24^.

^c^Excludes Bauer et al. (1986)^51^, Bauer et al. (1987)^52^, Turner et al. (1998)^42^, Barrett-Connor et al. (2005)^47^, Cauley et al. (2007)^49^, Sullivan et al. (2016)^10^, Chang et al. (2016)^21^.

^d^Excludes Bauer et al. (1986)^51^, Bauer et al. (1987)^52^, Baron et al. (1994)^38^, Karagas et al. (1996)^40^, Barrett-Connor et al. (2005)^47^, Tracy et al. (2006)^48^, Mackey et al. (2007)^50^, Sullivan et al. (2016)^10^, and Yusuf et al. (2020)^24^.

^e^Excludes Bauer et al. (1986)^51^, Bauer et al. (1987)^52^, Fisher et al. (1991)^54^, Griffin et al. (1992)^37^, Baron et al. (1994)^38^, Turner et al. (1998)^42^, Bohannon et al. (1999)^43^, Young et al. (2001)^44^, Cauley et al. (2008)^46^, Barrett-Connor et al. (2005)^47^, Tracy et al. (2006)^48^, Wright et al. (2012)^55^, Sullivan et al. (2016)^10^, Chang et al. (2016)^21^, and Berry et al. (2016)^22^.

**Table 3:** Relative risk of fracture associated with Hispanic ethnicity according to different exclusion criteria.

Studies included	Studies (*n*)	Relative risk (95% CI)	*p*
All studies	11	0.66 (0.55–0.79)	< 0.0001
Studies that used osteoporotic fracture as the outcome^a^	8	0.63 (0.48–0.82)	0.0007
Studies that controlled for various fracture risk factors^b^	6	0.74 (0.66–0.84)	< 0.0001
Studies with participants aged ≥ 65 years only^c^	4	0.73 (0.67–0.81)	< 0.0001
Studies with methodological quality score ≥ 7^d^	6	0.72 (0.66–0.79)	< 0.0001
Studies that used HR for effect size^e^	3	0.71 (0.69–0.74)	< 0.0001

CI = confidence interval.

^a^Excludes Cauley et al. (2007)^49^, Chang et al. (2016)^21^, and Yusuf et al. (2020)^24^.

^b^Excludes Bauer et al. (1986)^51^, Wright et al. (2012)^55^, Sullivan et al. (2016)^10^, Berry et al. (2016)^22^, and Yusuf et al. (2020)^24^.

^c^Excludes Bauer et al. (1986)^51^, Bauer et al. (1987)^52^, Turner et al. (1998)^42^, Barrett-Connor et al. (2005)^47^, Cauley et al. (2007)^49^, Sullivan et al. (2016)^10^, and Chang et al. (2016)^21^.

^d^Excludes Bauer et al. (1986)^51^, Bauer et al. (1987)^52^, Barrett-Connor et al. (2005)^47^, Sullivan et al. (2016)^10^, and Yusuf et al. (2020)^24^.

^e^Excludes Bauer et al. (1986)^51^, Bauer et al. (1987)^52^, Turner et al. (1998)^42^, Barrett-Connor et al. (2005)^47^, Wright et al. (2012)^55^, Sullivan et al. (2016)^10^, Chang et al. (2016)^21^, and Berry et al. (2016)^22^.

**Table 4:** Relative risk of fracture associated with Asian race according to different exclusion criteria.

Studies included	Studies (*n*)	Relative risk (95% CI)	*p*
All studies	9	0.55 (0.45–0.66)	< 0.0001
Studies that used osteoporotic fracture as the outcome^a^	6	0.50 (0.38–0.65)	< 0.0001
Studies that controlled for various fracture risk factors^b^	3	0.62 (0.52–0.73)	< 0.0001
Studies with participants aged ≥ 65 years only^c^	4	0.60 (0.52–0.70)	< 0.0001
Studies with methodological quality score ≥ 7^d^	5	0.60 (0.53–0.68)	< 0.0001
Studies that used HR for effect size^e^	3	0.66 (0.63–0.69)	< 0.0001

CI = confidence interval.

^a^Excludes Cauley et al. (2007)^49^, Chang et al. (2016)^21^, and Yusuf et al. (2020)^24^.

^b^Excludes Ross et al. (1995)^39^, Lauderdale et al. (1997)^41^, Wright et al. (2012)^55^, Sullivan et al. (2016)^10^, Berry et al. (2016)^22^, and Yusuf et al. (2020)^24^.

^c^Excludes Ross et al. (1995)^39^, Barrett-Connor et al. (2005)^47^, Cauley et al. (2007)^49^, Sullivan et al. (2016)^10^, and Chang et al. (2016)^21^.

^d^Excludes Ross et al. (1995)^39^, Barrett-Connor et al. (2005)^47^, Sullivan et al. (2016)^10^, and Yusuf et al. (2020)^24^.

^e^Excludes Ross et al. (1995)^39^, Barrett-Connor et al. (2005)^47^, Wright et al. (2012)^55^, Sullivan et al. (2016)^10^, Chang et al. (2016)^21^, and Berry et al. (2016)^22^.

**Table 5:** Relative risk of fracture associated with American Indian race according to different exclusion criteria.

Studies included	Studies (*n*)	Relative risk (95% CI)	*p*
All studies	6	0.80 (0.41–1.58)	0.5195
Studies that used osteoporotic fracture as the outcome^a^	4	0.72 (0.29–1.80)	0.4874
Studies that controlled for various fracture risk factors^b^	4	1.02 (0.93–1.11)	0.6894
Studies with participants aged ≥ 65 years only^c^	2	1.10 (0.99–1.22)	0.0769
Studies with methodological quality score ≥ 7^d^	4	1.06 (0.98–1.15)	0.1401
Studies that used HR for effect size^e^	2	1.03 (0.93–1.15)	0.5650

CI = confidence interval.

^a^Excludes Cauley et al. (2007)^49^ and Chang et al. (2016)^21^.

^b^Excludes Sullivan et al. (2016)^10^ and Berry et al. (2016)^22^.

^c^Excludes Barrett-Connor et al. (2005)^47^, Cauley et al. (2007)^49^, Sullivan et al. (2016)^10^, and Chang et al. (2016)^21^.

^d^Excludes Barrett-Connor et al. (2005)^47^ and Sullivan et al. (2016)^10^.

^e^Excludes Barrett-Connor et al. (2005)^47^, Sullivan et al. (2016)^10^, Chang et al. (2016)^21^, and Berry et al. (2016)^22^.

### Subgroup and sensitivity analyses for black people

In Black people, subgroup analyses by sex showed men (RR = 0.57, 95% CI = 0.51–0.63, *p* < 0.0001) had a higher risk of fracture than women (RR = 0.43, 95% CI = 0.39–0.47, *p* < 0.0001) when compared with their white counterparts. The risk of fracture was greater in studies that reported vertebral fractures (RR = 0.50, 95% CI = 0.36–0.68, *p* < 0.0001), were cohort in design (RR = 0.45, 95% CI = 0.42–0.48, *p* < 0.0001), were published before 2011 (RR = 0.46, 95% CI = 0.42–0.51, *p* <0.0001), and were adjusted for BMD (RR = 0.50, 95% CI = 0.42–0.58, *p* < 0.0001). However, these subgroup differences were not statistically significant due to overlapping confidence intervals within their own subgroups (Table 6). Substantial heterogeneity (I^2^ = 75%) was observed between the twenty-two studies. A sensitivity analysis was conducted by omitting one study in each analysis so as to determine its impact on heterogeneity. The study by Amir et al. was the single greatest contributor to the heterogeneity^23^. By removing this study, heterogeneity was reduced by 11% (I^2^ = 64%). The study methodology did not differ much compared to the other included studies, although it did contain the second-largest study population of all studies involving all races and ethnicities, and its population was composed strictly of individuals aged ≥ 65 in nursing homes. Regarding the pooled effect sizes, there were no major changes when each study was omitted one at a time.

**Table 6:** Subgroup analyses for the association between black race and risk of fracture.

Black people				
Subgroup	Studies (*n*)	Relative Risk (95% CI)	*p*	Between-group *p* value
Sex				
Women	14	0.43 (0.39–0.47)	< 0.0001	0.0004
Men	9	0.57 (0.51–0.63)	< 0.0001	
Anatomical site of fracture				
Hip	12	0.44 (0.40–0.48)	< 0.0001	0.37
Vertebrae	3	0.50 (0.36–0.68)	< 0.0001	
Study design				
Cohort	17	0.45 (0.42–0.48)	< 0.0001	0.92
Cross-sectional	4	0.40 (0.25–0.65)	0.0002	
Year of publication				
Before 2011	15	0.46 (0.42–0.51)	< 0.0001	0.48
After 2011	7	0.45 (0.41–0.49)	< 0.0001	
Adjusted for BMD				
Yes	3	0.50 (0.42–0.58)	< 0.0001	0.23
No	19	0.45 (0.42–0.48)	< 0.0001	

### Subgroup and sensitivity analyses for Hispanics

In Hispanics, subgroup analyses revealed the risk of fracture was greater in cross-sectional studies (RR = 0.74, 95% CI = 0.67–0.81, *p* < 0.0001), studies published before 2011 (RR = 0.67, 95% CI = 0.50–0.89, *p* = 0.0063), and studies that adjusted for BMD (RR = 0.89, 95% CI = 0.72–1.10, *p* = 0.2920). However, these subgroup differences were not statistically significant due to overlapping confidence intervals within their own subgroups (Table 7). Subgroup analyses by sex and fracture site were incomplete due to the lack of studies that reported the risk of fractures in men and the risk of vertebral fractures (Table 7). However, the RR of studies that reported the risk of fractures in women (RR = 0.59, 95% CI = 0.47–0.76, *p* < 0.0001) and risks of hip fractures (RR = 0.54, 95% CI = 0.38–0.76, *p* = 0.0006) did not statistically differ from the original association of Hispanic ethnicity with fractures (RR = 0.66, 95% CI = 0.55–0.79, *p* < 0.0001). There was considerable heterogeneity (I^2^ = 99%) observed between the studies included in the analysis. Another “one-study removed” sensitivity analysis revealed that the removal of Sullivan et al. moderately reduced heterogeneity (I^2^ = 66%)^10^. The study’s methodology did not differ significantly from the other included studies. However, it was the only included study whose entire study population resides in California. The study only controlled for age, while the majority of the other studies controlled for additional variables. The omission of Sullivan et al. changed the pooled effect size because its removal resulted in the relative fracture risk in Hispanics increasing to 0.73 (95% CI, 0.68–0.78)^10^.

**Table 7:** Subgroup Analyses for the Association Between Hispanic Ethnicity and Risk of Fracture.

Hispanics				
Subgroup	Studies (*n*)	Relative Risk (95% CI)	*p*	Between-group *p* value
Sex				
Women	8	0.59 (0.47–0.76)	< 0.0001	–
Anatomical site of fracture				
Hip	5	0.54 (0.38–0.76)	0.0006	–
Study design				
Cohort	6	0.68 (0.53–0.86)	0.0015	0.13
Cross-sectional	4	0.74 (0.67–0.81)	< 0.0001	
Year of publication				
Before 2011	5	0.67 (0.50–0.89)	0.0063	0.29
After 2011	6	0.66 (0.52–0.83)	0.0003	
Adjusted for BMD				
Yes	2	0.89 (0.72–1.10)	0.2920	0.049
No	9	0.62 (0.51–0.76)	< 0.0001	

### Subgroup and sensitivity analyses for Asian Americans

In Asian Americans, subgroup analyses showed the risk of fracture was higher in women (RR = 0.51, 95% CI = 0.40–0.65, *p* < 0.0001) and in studies published before 2011 (RR = 0.61, 95% CI = 0.51–0.74, *p* < 0.0001). Nonetheless, these subgroup differences were not statistically significant due to overlapping 95% CIs within their own subgroups (Table 8). Subgroup analyses by anatomical site of the fracture, study design, and adjustment for BMD were incomplete due to an insufficient number of studies (Table 8). However, the RR of studies that reported the risk of hip fractures (RR = 0.49, 95% CI = 0.35–0.68, *p* < 0.0001), were cohort studies (RR = 0.54, 95% CI = 0.44–0.67,* p* < 0.0001), and were not adjusted for BMD (RR = 0.56, 95% CI = 0.46–0.68, *p* < 0.0001) did not statistically differ from the original association of Asian race with fractures (RR = 0.55, 95% CI = 0.45–0.66, *p* < 0.0001). A similar considerable heterogeneity (I^2^ = 99%) was observed in the pooled effect sizes analysis. A sensitivity analysis demonstrated that the removal of Sullivan et al. minimally reduced heterogeneity (I^2^ = 83%)^10^. The study’s omission increased the relative risk of fracture in Asian Americans to 0.61 (95% CI, 0.54–0.68). Similar to studies included in the Hispanics analysis, Sullivan et al. was the only study that reported populations limited to California and that only adjusted the effect sizes for age^10^.

**Table 8:** Subgroup Analyses for the Association Between Asian Race and Risk of Fracture.

Asian Americans				
Subgroup	Studies (*n*)	Relative risk (95% CI)	*p*	Between-group *p* value
Sex				
Women	7	0.51 (0.40–0.65)	< 0.0001	0.68
Men	3	0.42 (0.30–0.60)	< 0.0001	
Anatomical site of fracture				
Hip	4	0.49 (0.35–0.68)	< 0.0001	–
Study design				
Cohort	8	0.54 (0.44–0.67)	< 0.0001	–
Year of publication				
Before 2011	4	0.61 (0.51–0.74)	< 0.0001	0.56
After 2011	5	0.53 (0.41–0.70)	< 0.0001	
Adjusted for BMD				
No	8	0.56 (0.46–0.68)	< 0.0001	–

### Subgroup and sensitivity analyses for American Indians

In American Indians, there were insufficient studies to properly conduct subgroup analyses by sex, anatomical site of the fracture, study design, year of publication, and adjustment for BMD (Table 9). In addition, none of the RR in the available subgroups statistically differed from the original association of the American Indian race with fractures (RR = 0.80, 95% CI = 0.41–1.58, *p* = 0.3436). In the pooled effect size analysis, there was also considerable heterogeneity (I^2^ = 99%). As in the previous groups, a sensitivity analysis was performed and determined the removal of Sullivan et al. completely removed heterogeneity (I^2^ = 0%)^10^. By omitting the study, fracture risk increased to 1.05 (95% CI, 0.98–1.14). Differences in study methodology between Sullivan et al. and the other included studies are similar to those observed in the other analyses^10^.

**Table 9:** Subgroup analyses for the association between American Indian race and risk of fracture.

American Indians				
Subgroup	Studies (*n*)	Relative risk (95% CI)	*p*	Between-group *p* value
Sex				
Women	4	0.68 (0.29–1.61)	0.3795	–
Anatomical site of fracture				
Hip	3	0.68 (0.23–1.99)	0.4837	–
Study design				
Cohort	5	0.76 (0.35–1.67)	.4993	–
Year of publication				
After 2011	6	0.80 (0.41–1.58)	0.5195	–
Adjusted for BMD				
No	5	0.79 (0.37–1.67)	0.5328	–

### Publication bias

**Figure 3:**
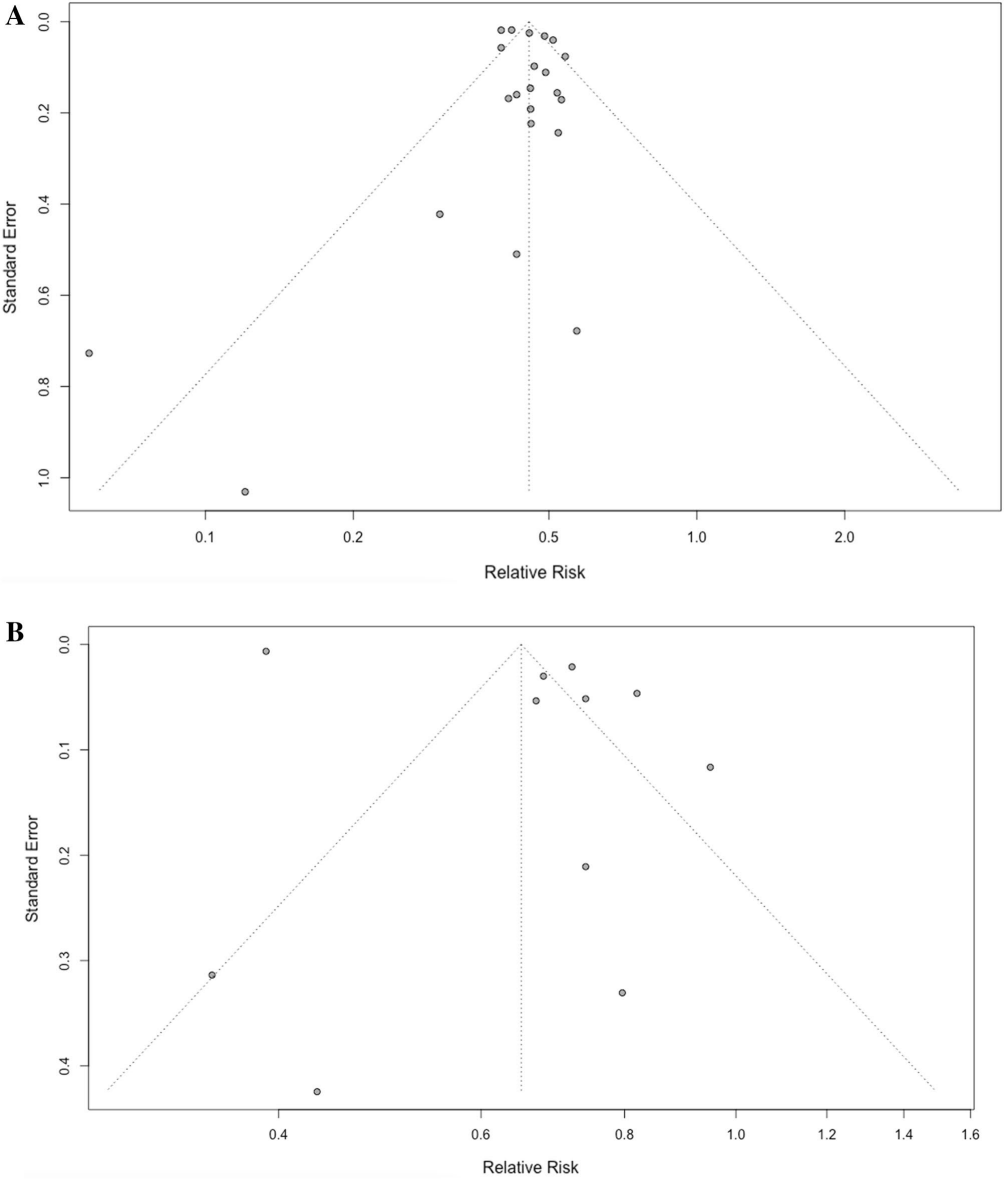
(**A**) Funnel plot of risk ratio versus standard error of relative risk in Black people. (**B**) Funnel plot of risk ratio versus standard error of relative risk in Hispanics.

Funnel plots and the Egger tests were performed to assess publication bias for Black people and Hispanics. We could not examine publication bias for Asian Americans and American Indians due to an insufficient number of studies (< 10). The funnel plots (Fig. 3) and Egger tests suggested there was no significant publication bias in Black people (*p* = 0.42); however, publication bias was detected in Hispanics (*p* = 0.036).

## Discussion

Our comprehensive meta-analysis of available observational studies examined the association between race and ethnicity and fracture risk. That analysis showed that people of racial-ethnic minority groups were at a lower fracture risk than white people. Compared with white people, the relative fracture risk decreased by 54% in Black people, 34% in Hispanics, 45% in Asian Americans, and 20% in American Indians.

Our study findings are consistent with previous literature reviews regarding the risk of fractures in different races and ethnicities^18,19^. However, there have not yet been any published systematic reviews or meta-analyses, and thus no quantitative data on the association between race and ethnicity and the risk of fractures. In the present study, we performed meta-analyses for each race group and included subgroup analyses and sensitivity analyses to further examine the robustness of our findings. In addition, while previous reviews explored data from countries outside the US, we focused strictly on studies conducted in the US population. Moreover, the previous reviews were published nearly a decade ago and could not integrate findings from recently published extensive studies^20–24^.

The role that race plays in clinical decisions has been an increasing point of discussion in recent years. In the context of osteoporotic fractures, a recent study by Vyas et al. has questioned the use of race in FRAX USA’s calculation of the 10-year probability of fractures^17^. However, the current study cements the idea of race and ethnicity as a clinical risk factor for fractures, similar to any other risk factors. While this supports the use of race adjustments in FRAX USA, it is important to consider patient demographics and other clinical risk factors when discussing predictions of fractures. Together, these factors can help properly identify individuals at high risk of fractures, guide clinicians to treat the appropriate patients, and help close the osteoporosis treatment gap^44^. Although the underlying difference in risk factors between separate race groups is not yet fully understood, several risk factors have been heavily implicated in the risk of osteoporosis and fractures. While risk factors such as smoking, alcohol consumption, and Type 1 diabetes have been shown to increase the risk of fractures, low BMD continues to be one of the strongest predictors of future fractures^45–47^. In Black people, subgroup analysis by studies that controlled for BMD revealed the association between race and fracture risk continued to be significant even after adjusting for BMD. However, a similar conclusion cannot be made for the other races and ethnicities due to statistically insignificant results or a limited number of studies that adjusted for BMD. A fact is that for every standard deviation decrease in femur neck BMD (FNBMD), the risk of hip fractures increases by 294% in men and 288% in women at the age of 65^48^. Previous studies have continuously reported higher BMD in Black people in comparison with the other races and ethnicities^21,22,24^. Black people also exhibit a decreased age-adjusted annual decline in BMD^49^. The overall higher BMD at all sites and lower BMD loss as Black people age may help explain their significantly lower risk of fractures. Another explanation that may influence the measured difference between the risk of fractures in Black and white people is the difference in life expectancy at birth between the two races. In the US, Black people have a life expectancy at birth of 72 years, whereas white people have a life expectancy of 78 years^50^. As osteoporosis and resulting fractures are most common in the elderly population, with higher age groups at increasing risk, differences in life expectancy can influence the availability of Black participants in observational studies, thus, underestimating the rates and risks of fractures. Examining data from other countries with diverse but distinct racial and ethnic groups revealed similarities in the risks and rates of fractures between white and Black populations. For example, in South Africa, the African population was observed to possess the lowest incidence rates of fractures. The white population had the highest incidence rates, while the other races and ethnic groups were between white people and Africans^51^. These observed differences show similarity to the situation in the US. Consequently, the latest version of FRAX incorporated a South African-specific model in which race adjustments are performed in the tool’s output.

In Hispanics, FNBMD measurements are higher than in white people but lower than in Black people. In NHANES 2005–2006, FNBMD was 6.6% greater in Mexican American women than in white women, while FNBMD in Black women was 10% greater than in white women^52^. MrOS demonstrated FNBMD was 2% higher in Hispanic men than in white men^53^. Interestingly, several large studies have shown lumbar spine BMD (LSBMD) in Hispanics to be lower than in white people^54–56^. Unfortunately, we were unable to explore how this translates into vertebral fracture risks due to the limited number of studies reporting vertebral fractures in Hispanics. In American Indians, data regarding BMD measurements have been scarce. While our meta-analysis showed American Indians were at a decreased risk of fractures compared to white people, the wide confidence interval suggests no statistically significant difference in fracture risk between the two races. Data from the WHI study and the National Osteoporosis Risk Assessment study showed American Indian women possess similar BMD at various sites in comparison to white women^23,57^. In contrast to the relation between BMD and fracture risk observed in the other races, Asian Americans possess lower BMD than white people, yet are at a decreased risk of fractures. There have been various attempts to address this paradox. One explanation contributes to the lower risk of fractures in Asian Americans to their skeletal geometry, whereby Asian Americans tend to have a shorter hip axis length^58,59^. Since longer hip axis length has been associated with an increased risk of fractures, the shorter hip axis length in Asian Americans may confer protection against hip fractures^60,61^. Another explanation proposes studies that reported areal BMD (aBMD) did not adjust for weight, height, and other important covariates. An example of this was seen in the Study of Women’s Health Across the Nation. Compared with Black and white women, Asian American women seemed to possess the lowest unadjusted lumbar vertebrae and femoral neck aBMD^62^. However, after adjusting for weight and other covariates, aBMD was greater in Asian American women than in white women. Similarly, a study on men from four countries found that the aBMD gap between white people and Asians was significantly reduced after adjusting for height and weight^54^.

Although these factors could help explain the racial and ethnic differences in fracture risks, it must be noted that many of these factors, such as aBMD, were not adjusted in the analyses of the included studies. As seen from the subgroup analyses, most included studies did not adjust for BMD. Only 3 of 21 studies in Black people and 2 of 11 studies in Hispanics adjusted for BMD, while no studies in Asian Americans and American Indians adjusted for BMD^20,33–35^. Furthermore, other well-established risk factors for fractures known to differ between races and ethnicities, such as volumetric BMD (vBMD) or failure load, have not been included as a confounder in the included studies’ analyses^63,64^. There are also socio-demographic and socioeconomic determinants of fracture risks, such as education and income. For example, an inverse relationship between fracture risk and income was observed in Medicare beneficiaries^65^. In our included studies, only 4 of 22 studies adjusted for education, and only one adjusted for family income^20,31,35,37^. These clinical, socio-demographic, and socioeconomic factors all play an important role in the risks of fractures. They should be considered when discussing the fracture risks between races and ethnicities. Future studies should explore these factors further in-depth to help explain the observed variations in risks and rates of fracture among different races and ethnicities.

While our findings suggest that a focus should be placed on white people and perhaps American Indians, it is crucial to understand that the number of fractures and associated costs is expected to balloon over the coming years due to shifting demographics in the US. While this can be attributed to an overall aging population, we must also consider that population growth in minority races exceeds that of white people^14^. Thus, the significance of proper bone health should not be neglected for all population groups. Healthcare disparities exist in all stages of care, from screening to post-fracture outcomes. In studies of Black women, they were less likely than their white counterparts to be referred for dual-energy x-ray absorptiometry (DXA) screening, to know their DXA results, and to be properly prescribed osteoporosis medication^11,66,67^. In a large study of Medicare patients with hip fractures, Black and Hispanic women were 48% and 34%, respectively, less likely than white women to have undergone bone testing prior to their fractures^68^. However, data regarding screening rates in races and ethnicities other than Black have been scarce and mixed, making it harder to draw conclusions^69^, which demonstrates the need for more research on the disparities in the screening and treatment of minority groups. With regards to post-fracture care, outcomes similarly differ by race and ethnicity. After suffering common osteoporotic fractures, such as hip or vertebral fractures, the risk of mortality is not the same in every race. In a study of US Medicare data from 2010 to 2016, Black women suffered greater rates of mortality, frailty, and poverty after a fracture when compared to white women^70^. In another large study of three cohorts, white people were 1.74 more likely to survive six months post-fracture than other races and ethnic groups, including Black people and Hispanics^7^. The reasons for these discrepancies are not yet fully understood. The current research adds to the pool of knowledge that physicians and other healthcare providers can use when assessing fracture risk in patients of different races. Our research is, to our knowledge, the first study that provides quantitative evidence regarding the association between race and ethnicity and the risk of fractures. However, as evidenced by the discrepancy in the number of studies that reported results for Black people and other races and ethnicities, there is an obvious need for more research into Hispanics, Asian Americans, and American Indians. In addition to including more participants of these races and ethnicities, future research should also attempt to adjust for both age and BMD, as these are important risk factors for developing osteoporosis and subsequent fractures. Lastly, there is an urgent need for more research into sociological factors influencing socioeconomic and socio-demographic characteristics of different racial and ethnic minority groups and how these interplay with the risk of osteoporosis and osteoporotic fractures, which is essential in reducing the observed healthcare disparities across different racial and ethnic groups.

Our study has several limitations. First, there was substantial to considerable heterogeneity in all of the analyses. In three of four analyses, it may be possible to attribute this to the nature of *I*^2^. When the number of studies pooled together is small, there is a tendency for I^2^ to introduce significant bias^71^, which may partially explain the heterogeneity observed in the Hispanic, Asian American, and American Indian analyses (I^2^ = 99%). Another explanation of heterogeneity may be pooling cross-sectional studies with other study designs. Although cross-sectional studies are less expensive and more convenient to perform than case-control or cohort studies, they are often more susceptible to bias, such as non-response and recall bias^72^. Subgroup analyses by study design were performed to ensure the pooling of different study designs did not significantly influence our findings. In the subgroup analyses by study design for Black people and Hispanics, no significant differences in effect sizes were observed when comparing cross-sectional and cohort studies. In Asian Americans and American Indians, subgroup analyses were not performed due to the limited studies available. Another explanation could be due to the inherent nature of pooling observational studies. Oftentimes in such meta-analyses, it is difficult to control for the baseline characteristics of participants across the included studies, especially given the number of variables that can influence the risk of osteoporotic fractures. Second, our meta-analyses pooled several measures of effect sizes together (OR, RR, and HR). As previously mentioned, OR is often interpreted as broadly equivalent to RR due to the rare nature of fractures^24^. In contrast, HR differs from RR because it considers the timing of the outcome. Nonetheless, HR has been deemed broadly equivalent to RR, and pooling the two is common in meta-analysis research^73–76^. To ensure the addition of HR did not affect our results, we performed a sensitivity analysis by including only studies that reported effect sizes using HR. In analyses where sufficient studies were available, the exclusion of RR/OR did not considerably affect our findings, which remained significant. Third, it is important to consider socio-demographic, socioeconomic, and other clinical risk factors when discussing fracture risks in different races and ethnicities. In our meta-analysis, most included studies did not account for many of these established confounders known to influence fracture risks. Finally, our research focused on race and ethnic groups within the US only and thus may not be generalizable to other countries. Furthermore, it should also be taken into consideration that race and ethnic groups in the US are comprised of individuals with ancestry from different countries with varying fracture rates. This may translate into differing risks of fractures for individuals within the same race and ethnic group and is why patient demographics and other clinical risk factors, along with race and ethnicity should be considered when identifying patients at high risk of fractures.

## Conclusion

Our study showed that people of other races and ethnicities in the US are at a lower risk of fracture than white people. This decrease in risk was most significant in Black people and least significant in American Indians; a moderate decrease in risk was observed in Hispanics and Asian Americans. Our findings add to the tools available for healthcare providers who screen, diagnose, and treat men and women at risk of fractures. The work we have done provides quantitative data regarding fracture risk across different racial and ethnic groups, which along with patients’ clinical information, helps identify those who would benefit most from the initiation of osteoporosis treatment and help close the osteoporosis treatment gap. Our work also demonstrates the need for further research into fracture risks and their contributors. There are substantial gaps in osteoporosis research, especially in Hispanic, Asian American, and American Indian populations, and the currently available research clearly demonstrates the presence of healthcare disparities in minority populations.

### Methods

This study was conducted in accordance with the Preferred Reporting Items for Systematic Reviews and Meta-Analyses (PRISMA) guidelines and the Meta-Analysis of Observational Studies in Epidemiology (MOOSE) guidelines^77,78^. The protocol of this review was prospectively registered on PROSPERO (CRD42021239943).

### Search strategy and data sources

A comprehensive search of PubMed and EMBASE electronic databases was performed to include studies from the databases’ date of inception to October 20, 2021. An updated literature search was conducted on December 23, 2022. The search strategy for each database is reported in Supplemental Table S1 and S2. References from relevant studies were also searched to identify other potentially eligible studies. For this meta-analysis and review, studies were limited to those published in English.

The following search terms were used to identify studies: “fractures,” “blacks,” “African American,” “whites,” “Caucasian,” “Hispanic,” “Latino,” “Asian,” “Native American,” “American Indian,” “Alaska Native,” “Pacific Islander,” “Native Hawaiian.”

### Study selection

In the initial study selection stage, investigators Y.B. and Z.L. independently screened each article’s title and abstract from the electronic literature search for studies that investigated the association between race and ethnicity and the risk of fractures. The following criteria were used to screen for relevance: (1) the study population was limited to the US, (2) the study reported the effect size of racial-ethnic minority groups using white people as the reference group, and (3) the study reported fractures as the outcome. We defined fractures as those occurring in any site. However, fractures attributed to major trauma were excluded. Citations deemed irrelevant by both investigators were excluded, and articles with disagreements at the screening were included for a full review in the second study selection stage. There are no clinical trial studies that meet the inclusion criteria.

In the second study selection stage, the full text of each article obtained during the initial study selection stage was reviewed and evaluated for inclusion. Studies had to be cohort, cross-sectional, or case-control in design, had to report outcomes using odds ratio (OR), relative risk (RR), or hazard ratio (HR), and had to include the corresponding 95% confidence intervals (CI). Studies were also included if the effect sizes were calculable from the provided data. Disagreements between investigators were resolved through discussion, and if necessary, a third investigator (Y.X.) was consulted. Agreement between investigators was evaluated using the κ statistic, a robust measure of inter-rater reliability.

### Data extraction and study appraisal

Investigators Y.B. and Z.L. performed data extraction independently. The following information was extracted from each study: study characteristics (title, name of first author, year of publication, journal, duration of follow-up in cohort studies, number of cases and controls in case-control studies, total number of study participants), participants’ characteristics (age, sex, and race and ethnicity), outcomes and ascertainment of outcomes, and risk estimates (adjusted RR, OR, and HR and 95% CI). For cases of missing or unclear data, study authors were contacted for clarification and/or additional data. In our systematic review and meta-analysis, we refer to the biological sex when referring to men and women. Race and ethnicity are in accordance with the United States Census Bureau, whereby white people, Black people, Asian Americans, and American Indians are race categories and Hispanic ethnicity. The methodological quality of both the case-control and cohort studies was assessed using the Newcastle-Ottawa Scale (NOS)^79^. For cross-sectional studies, a modified NOS was used. In accordance with MOOSE guidelines, quality scores were not used as weights in the analyses. Instead, they were used in the sensitivity analysis, where studies with low scores were excluded. A study was considered high-quality if it scored ≥ 7 on the NOS, while a low-quality study scored < 7.

### Statistical analysis

In our meta-analysis, RR was used to measure the association between race and ethnicity and the risk of fracture. We calculated the pooled effect size using OR, RR, and HR and the 95% CIs reported by the included studies. Due to the occurrence of fractures being rare, we approximated ORs as RRs^80^. When determining the weights of the studies, the inverse-variance method was used.

Between-study heterogeneity was measured using the Higgins *I*^*2*^ index, which measures how much of the variability in the effects is due to heterogeneity instead of chance alone^81^. We interpreted I^2^ < 40% as minimal heterogeneity, 30–60% as moderate heterogeneity, 50–90% as substantial heterogeneity, and > 75% as considerable heterogeneity^82,83^. In light of the heterogeneity, the DerSimonian and Laird random-effects model was used to pool the overall effect sizes^84^.

Sensitivity analyses were performed to assess the robustness of our findings. We examined the influence of race and ethnicity by fracture definition, adjustment for age and other fracture risk factors, stratification by age (age ≥ 65 vs. < 65), methodological quality score, and type of effect size. Pre-specific subgroup analyses were also conducted to determine if study demographics influenced the effects of race and ethnicity on the risk of fractures. The subgroup analysis variables were sex (men vs. women), anatomical site of fracture (hip vs. vertebrae), study design (case-control, cross-sectional, or cohort), year of publication (pre-2011 vs. post-2011), and adjustment for BMD.

Potential publication bias was examined by constructing a funnel plot that plotted RRs against their standard errors^85^. The Egger’s test was also conducted to help assess the presence of publication bias in the funnel plots^86^. For races and ethnicities with less than ten studies, a funnel plot was not performed because its ability to detect publication bias through asymmetry is too unreliable^87,88^.

All data analyses were conducted using the R statistical software (Version 4.0, Core Team, Vienna, Austria). A *p*-value of 0.05 or less was considered to be statistically significant.

## Supplementary Information

Supplementary Information.

## Data Availability

All data generated or analyzed during this study are included in this published article.
